# FGF treatment of host embryos injected with ES cells increases rates of chimaerism

**DOI:** 10.1007/s11248-016-9997-6

**Published:** 2016-11-21

**Authors:** Cathérine Dupont, Friedemann Loos, John Kong-A-San, Joost Gribnau

**Affiliations:** Erasmus MC, Rotterdam, The Netherlands

**Keywords:** Transgenesis, Blastocyst injection, Chimaera, FGF4, Tetraploid complementation, ES cell

## Abstract

In spite of the emergence of genome editing tools, ES cell mediated transgenesis remains the most controllable way of creating genetically modified animals. Although tetraploid (4N) complementation of 4N host embryos and ES cells, is the only method guaranteeing that offspring are entirely ES cell derived, this technique is challenging, not always successful and difficult to implement in some laboratory settings. The current study shows that pretreatment of host blastocysts with FGF4 prior to ES cell injection can provide an alternative method for the generation of animals displaying high rates of chimaerism. Chimaerism assessment in E11 fetuses and born pups shows that a large percentage of resulting conceptuses show a high ES cell contribution from implantation onwards and that developing pups do not necessitate c-section for delivery.

## Introduction

The capacity of murine embryonic stem (ES) (Evans and Kaufman 1981; Martin 1981) and induced pluripotent stem (iPS) cells to contribute to all embryonic lineages when injected into murine pre-implantation embryos, has had a tremendous impact on transgenic research (Bradley et al. 1984). Over the years the technique has been adapted in order to increase the efficiency of transgenesis. The first improvement came in 1990 when Nagy et al. aggregated ES cells with tetraploid embryos (Nagy et al. 1990). As previous investigators had shown that the viability of tetraploid cells in pups is poor (Kaufman and Webb 1990; Lu and Markert 1980; Snow 1975), the assumption was that resulting offspring from an aggregation between tetraploid and ES cells would be merely derived from the ES cells. While initially there were no viable offspring, a later study improved the method by utilizing low passage number inbred ES cells (Li et al. 2007; Nagy et al. 1993). The researchers (Nagy et al. 1993) hypothesized, however, that not only the efficiency might be dependent on the passage number but also on the background of the ES line (inbred or hybrid). Eggan and colleagues confirmed this latter hypothesis and showed that hybrid ES cell lines were more potent in generating live offspring than inbred ES cell lines (Eggan et al. 2001). Nevertheless the efficiency of tetraploid complementation assays remains rather unsatisfying as the technique only works with certain ES cell lines and pups not only need to be delivered via c-section requiring additional nursing mothers but are also subject to various lethal disorders. As an alternative to tetraploid complementation, injection of ES cells into pre-compaction stage embryos has been introduced (Poueymirou et al. 2007). This technique permits the generation of highly chimaeric mice with less technical hurdles, but so far has not always been successful in different laboratory settings (Hu et al. 2013). The direct application of genome editing tools in oocytes (Shen et al. 2014; Singh et al. 2015; Wang et al. 2013) has briefly led to believe that ES cell mediated transgenesis would become needless. The difficulty to create mice with conditional or compound mutations via direct application of these genome editing tools in oocytes (Singh et al. 2015), however, stillhighlights the importance of ES cell mediated transgenesis.

The success of creating animals that are entirely ES cell derived through tetraploid complementation rests on the limited developmental potential of the epiblast from tetraploid host embryos. In normal circumstances, cells from the inner cell mass (ICM) differentiate to become either the primitive endoderm or the naïve epiblast. Cell fate determination in the ICM is governed by FGF signaling as FGF stimulates the formation of primitive endoderm (Yamanaka et al. 2010). Inhibition of FGF signaling in preimplantation embryos is therefore commonly utilized to derive ES cells (Gallagher et al. 2003). The current study shows that the contribution of ES cells injected into preimplantation embryos can be improved if the developmental potential of the epiblast of the host embryo is biased towards primitive endoderm following exposure to high concentrations of FGF4.

## Materials and methods

### Embryo collection

Mice were exposed to an artificial 12 h light–dark cycle (with lights on from 7:00 to 19:00 h). The ovarian cycle of females (all strains, age between 6 and 10 weeks) was synchronized by intra-peritoneal injections of Folligonan and Chorullon (50U/ml, both one injection with 48 h in between, usually around 13.30 pm). Dosage of the hormones varied according to the age and strain of the females (Cast/Eij: 80–100 µl; all other strains:100 μl if 6 weeks, 125 μl if 7 weeks, 150 μl for females 8 weeks and older). Following the last injection each female mouse was mated with a male mouse. About 20 h after the last injection, mating was evaluated by looking for the presence of a copulation plug in the vagina. Females that had mated were euthanized at E2.5 to collect host embryos for ES cell injection or at E3.5 for ES cell derivation.

### ES cell derivation

ES cell lines were derived from both inbred and hybrid F1 blastocysts. Embryos for ES cell derivation were obtained from female and male mice from strains C57BL/6JOlaHsd (Envigo, The Netherlands), 129S2/SvHsd (Envigo, The Netherlands) and Cast/Eij (Jackson, USA). In order to derive ES cells with a visual marker, we preferably utilized mice of the C57BL/6JOlaHsd (Envigo, The Netherlands) strain carrying an Actin-GFP transgene (Okabe et al. 1997). E3.5 blastocysts were placed into culture dishes coated with gelatin (0.2%) and irradiated Mouse Embryonic Fibroblasts (MEFs, density 3×10^4^/cm^2^) in 2i ES cell medium containing Dulbecco’s modified eagles medium DMEM (Lonza, BE12-604F/U1), 15% Fetal Calf Serum (FCS) (Hyclone), 1% PenStrep (PS) (Life Technologies), 1 mM non-essential amino acids (NEAA) (Lonza), 50 mM β-mercaptoethanol (Life Technologies), 20 ng/ml leukaemia inhibitory factor (LIF) (homemade), MEK inhibitor (Cell Signalling, PD98059, 4 µM final) and GSK3inhibitor (Stemgent, CHIR99021, 3.3 µM final). Approximately one week after embryo recovery, the outgrowth of the ICM was picked and placed in pre-warmed (37 °C) 0.25% Trypsin/EDTA (Life Technologies) droplets for 5–10 min and following harsh pipetting replated in the same culture conditions as described above. Once the ES cell lines were stable following enzymatic passaging (generally two to five passages) using pre-warmed (37 °C) 0.25% Trypsin/EDTA, ES cells could be propagated in cell culture dishes coated with gelatin (0.2%) and irradiated Mouse Embryonic Fibroblasts (MEFs, density 3×10^4^/cm^2^) in ES cell medium without GSK3 inhibitor and MEK inhibitor. To avoid the presence of MEFs in ES cell suspensions prepared for blastocyst injection, however, ES cells were prior injection into blastocysts cultured without MEFs for 1–2 passages on gelatin (0.2%) coated cell culture dishes in ES cell medium containing GSK3 and MEK inhibitor.

### Host embryos for ES cell injection (Fig. 1)

Host embryos were obtained from inbred crosses of C57BL/6JOlaHsd (Envigo, The Netherlands) mice. Embryos were flushed from the oviducts at E2.5 and randomly placed into pre-equilibrated (approximately 2 h) mineral oil covered culture drops containing either KSOM or KSOM supplemented with FGF4 (1000 ng/ml, Sigma) and heparin (1 µg/ml, Sigma) and kept in culture at 37 °C and 5% CO_2_. The following day, at E3.5, 15–25 ES cells were injected into the blastocoel of cavitated embryos. The embryos were placed back into their respective culture drops and transferred if a blastocoel reformed. Approximately 5–10 embryos were transferred to the uterine horns of pseudopregnant (cross between CBA/J from Charles River Laboratories, The Netherlands and C57BL/10ScOlaHsd.from Invigo, The Netherlands) either the same day within a few hours (E3.5) or early in the morning of the day following (E4.5) the blastocyst injection. The pseudoporegnant females underwent general anaesthesia using an intra-peritoneal injection of Ketamine (120 mg/kg) and Rompun (7.5 mg/kg) and subsequently received a pain killer (Rimadyl Cattle 5 mg/kg, subcutaneous). Uteri were exposed through a small incision on the back of the mouse and embryos were mouth pipetted into the uteri through an opening made with a needle. Following the transfer, uteri were pushed back in the abdomen and a suture was made around the incision. To prevent a drop in body temperature during wakening, the mice were placed on a heated mat.

**Fig. 1:**
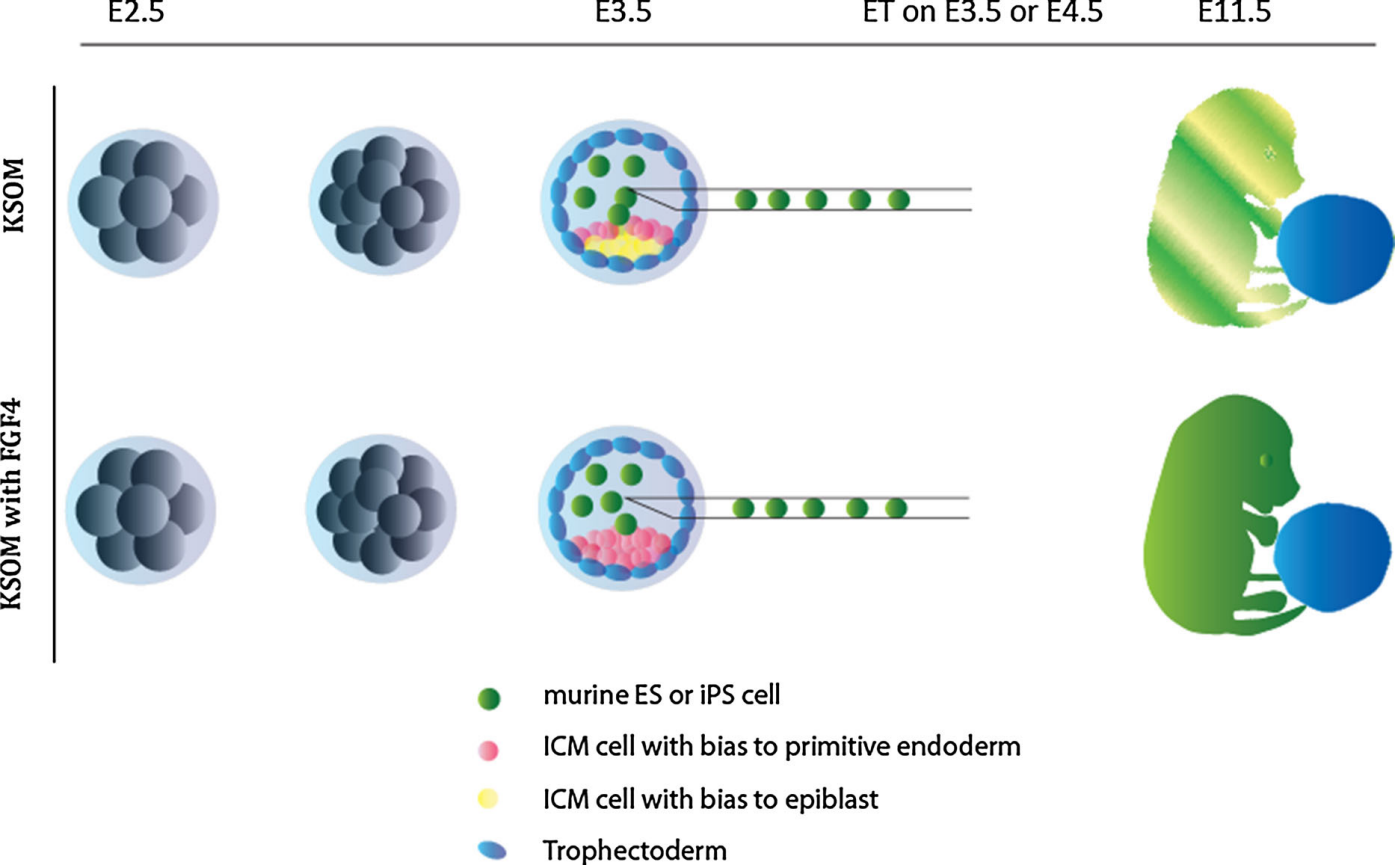
Schematic representation of the utilized method. Embryos were flusded at E2.5 around compaction and were immediately placed into either regular KSOM or KSOM supplemented with FGF4 (1000 ng/ml) and heparin (1 µg/ml). At E3.5, blastocysts were injected with ES cells (inbred or hybrid) and placed into their respective culture medium. Embryos that reformed the blastocoel were transferred to the uterine cavity either the same day (E3.5) of the injection or the following day (E4.5)

### FACS analysis of E11 fetuses

E11 fetuses were removed from the uterine horns and dissociated to single cells by cutting each fetus into small pieces, exposing the pieces to pre-warmed (37 °C) 0.25% Trypsin/EDTA (Life Technologies) for 10 min followed by their aspiration into 2 ml syringes using needles of various diameter. The suspension (approximately 2 ml) was subsequently collected in a tube containing 3 ml of PBS with 20% FCS in order to inactivate the 0.25% Trypsin/EDTA. Following centrifugation (1000 rpm for 5 min), the pellet was dissolved into 1 ml of PBS with 2% FCS and passed through a cell strainer before the percentage of GFP positive cells was assessed via FACS.

### GFP contribution in adult offspring

Lungs, heart and brain representing respectively endoderm, mesoderm and ectoderm were dissected from adult offspring (age over 30 weeks) with a chimaeric coat colour and the contribution of GFP relative to H2A was assessed via real-time PCR. Non-chimaeric offspring were not included in this analysis. Genomic DNA was extracted via a phenol chlorophorm extraction and DNA was precipitated through a standard ethanol precipitation. For the genotyping PCR, all samples were analyzed were run in a 10 μl final reaction volume using the BioRad CFX 384 Real-time System. The reaction mixture contained 2× SYBR Green PCR Master Mix (Applied Biosystems, #4309155), primer (either GFP or H2A) and 2 μl of genomic DNA (approximately 10 ng). The sequence of the primers were as follows: H2A Forward primer CTTTGCGCTTTCGTGATGTC, H2A Reverse primer CCCCACCGGGAACTGTAG, GFP Forward primer CCTACGGCGTGCAGTGCTTCAGC GFP Reverse primer CGGCGAGCTGCACGCTGCGTCCTC. After an initial hold at 95 °C for 10 min, reaction mixtures underwent 40 cycles of 30 s at 95 °C, 30 s at 57 °C and 1 min at 72 °C. Standard curves were generated using mouse ES cell genomic DNA from an Actin GFP ES cell line and efficiencies used to calculate the abundance of GFP.

## Results

### E11 chimaerism rate improved following exposure to FGF4

**Fig. 2:**
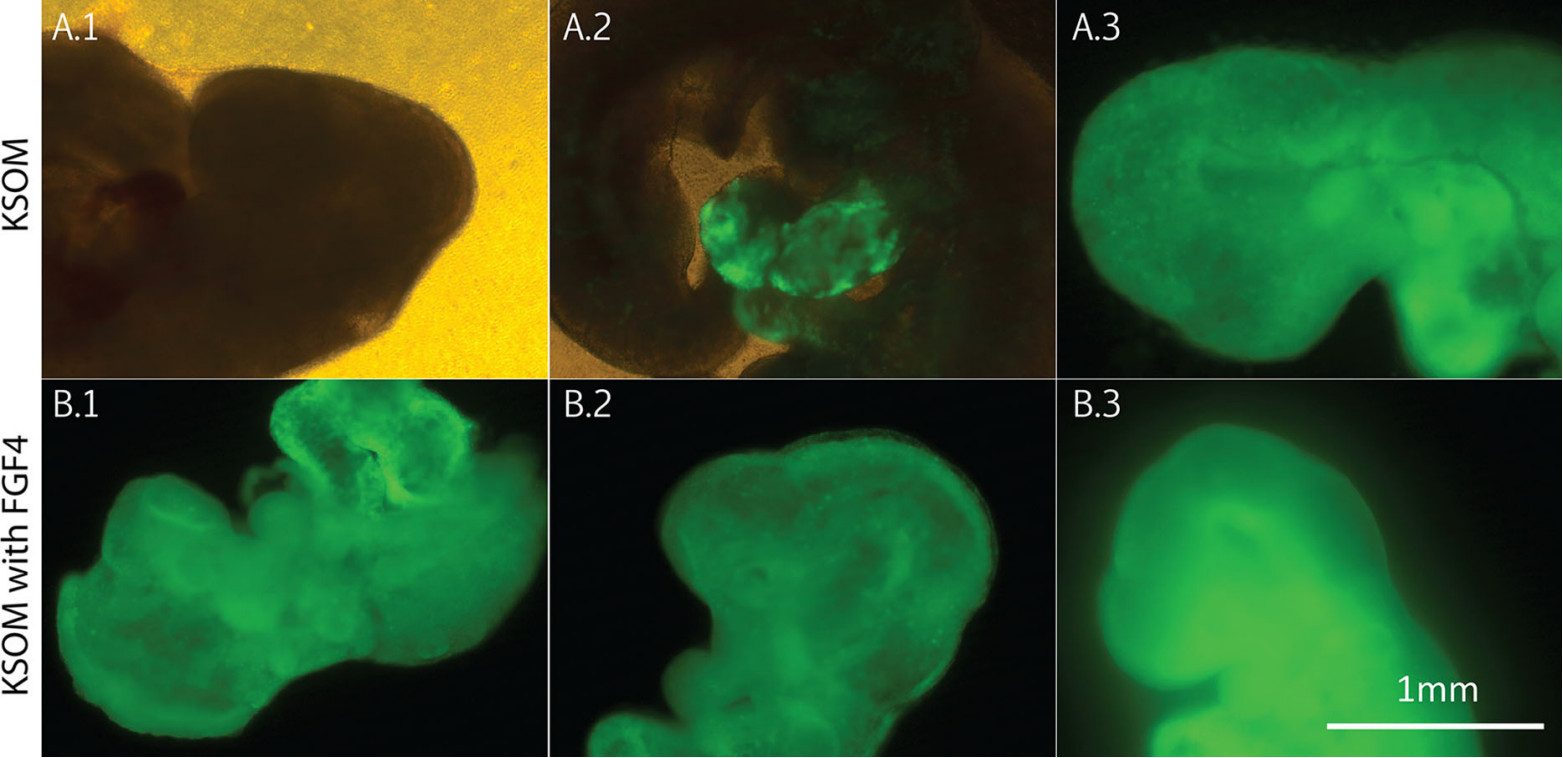
Representatitve fluoresecent pictures of E11 fetuses. The top panel represents fetuses grown in KSOM prior ES cell injection, whereas the bottom panel represents fetuses derived from preimplantation embryos that were cultured in KSOM supplemented with FGF4 (1000 ng/ml) and heparin (1 µg/ml) prior ES cell injection. A1, A3, B1, B2 and B3 show the head of fetuses that are approximately of the same size. A2 shows chimaerism in front legs of a larger fetus

**Fig. 3:**
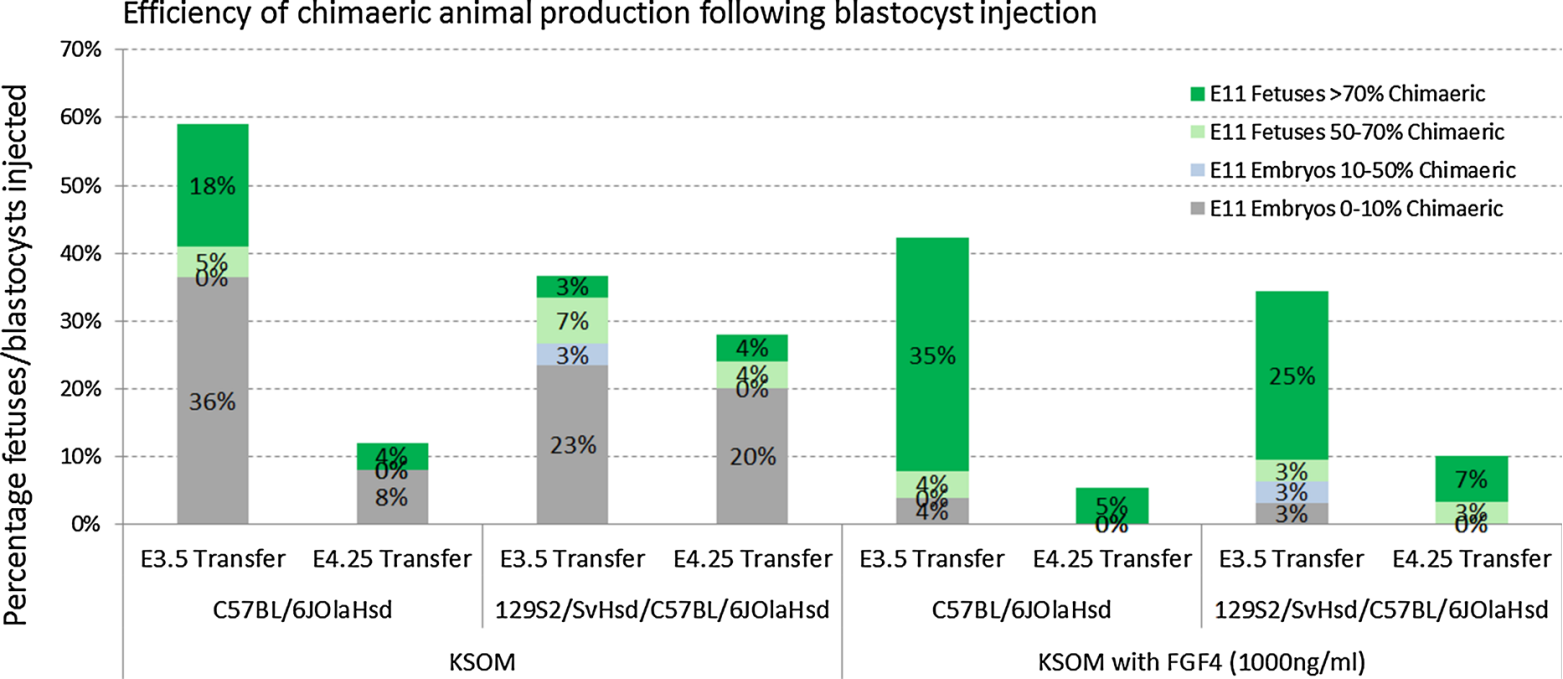
Chimaerism in E11 fetuses. Shown is the number of fetuses presented in groups with percentages of GFP+ cells assessed by FACS analysis ranging from 0 to 10, 10 to 50, 50 to 70 and 70 to 100% relative to the total number of E3.5 blastocysts injected

ICM cells of preimplantation embryos exposed to high concentrations of FGF4 and heparin are forced towards development of extra-embryonic lineages (Yamanaka et al. 2010). This study indicated that ICM cells are entirely biased to develop towards the primitive endoderm when the FGF4 concentration approximates 1000 ng/ml. To test whether the reduced developmental fate of host embryos exposed to FGF4 (1000 ng/ml) and heparin (1 µg/ml) is beneficial for the production of chimaeric mice, E11 fetuses created following injection of ES cells into host embryos, that either were or were not exposed to FGF4 and heparin were analyzed. Embryos were recovered at E2.5 and placed in culture. The following day, embryos had attained the morula or blastocyst stage. Only blastocysts were injected with ES cells and were re-placed into their respective culture medium. Both inbred C57BL/6JOlaHsd and hybrid 129S2/SvHsd/C57BL/6JOlaHsd Actin GFP ES cell lines were injected into blastocysts cultured in regular KSOM or KSOM supplemented with FGF4 (1000 ng/ml) and heparin (1 µg/ml). In total, respectively, 52 and 58 blastocysts, were transferred on the same day of ES cell injection; whereas 75 and 86 blastocysts were transferred in the morning of the day following the blastocyst injection. At E11, fetuses were recovered (Fig. 2) and dissociated (mechanically and enzymatically) to single cells. Four Actin-GFP and wildtype fetuses created via natural conception were included during FACS analysis in order to determine which percentage of GFP+ cells would be representative of a fully ES cell derived fetus. Whereas the negative controls display no GFP+ cells, the percentage of GFP+ cells in positive controls obtained through natural mating from an Actin-GFP male with a wildtype female varied from 70 to 80%. For E3.75 embryo transfer (ET) fetuses, 37.5% of the fetuses derived from ES cell injected embryos cultured in regular KSOM showed contribution of the injected ES cells. In contrast, 90.9% (χ^2^, *p* value <0.0002) of the fetuses generated by injection of ES cells in host blastocysts cultured in KSOM supplemented with FGF4 (1000 ng/ml) and heparin (1 µg/ml) were chimaeric (Table 1). Since Actin-GFP fetuses obtained through natural mating display GFP rates ranging from 70% and up, 20.8 and 77.7% (χ^2^, *p* value <0.00012)of the fetuses were entirely composed of injected ES cells when derived from preimplantation embryos cultured in respectively regular KSOM or KSOM with FGF4 and heparin. Comparable values could be observed for E4.5 ET fetuses, although, the implantation efficiency per transferred blastocyst on E4.5 was extremely reduced. Whereas a statistical difference was also observed for the number of chimaeric fetuses (χ^2^, *p* value <0.005) and the number of fetuses entirely derived from ES cells (χ^2^, *p* value <0.013) following a transfer on day 4.25 (4.25 ET), the efficiency to obtain a fetus at E11 per injected blastocyst, was too low to implement day 4.25 transfers (Fig. 3).

**Table 1:** Rate of chimaerism measured by percentage GFP cells through FACS analysis in whole E11 Fetuses

Strain ES cell	Treatment	Embryos tranfer	Total Injected embryos transferred	Total E11 fetuses	E11 fetuses chinmaeric >10%	E11 fetuses 10–50%	E11 fetuses 50–70%	E11 fetuses >70%
C57BL/6JOlaHsd	KSOM	Day3.5 ET	22	13 (59.1%)	5 (22.7%) ((38.5))	0 (0%) ((0%))	1 (4.5%) ((7.7%))	4 (18.2%) ((30.8%))
		Day4.5 ET	50	6 (12%)	2 (4.0%) ((33.3%))	0 (0%) ((0%))	0 (0%) ((0%))	2 (4%) ((33.3%))
129S2/SvHsd/C57BL/6JOlaHsd		Day3.5 ET	30	11 (36.7%)	4(13.3%) ((36.4%))	1 (3.3%) ((9.1%))	2 (6.7%) ((18.2%))	1 (3.3%) ((9.1%))
		Day4.5 ET	25	7 (28%)	2 (8.0%) ((28.6%))	0 (0%) ((0%))	1 (4%) ((14.3%))	1 (4%) (14.3%))
		Total E3.5 ET	52	24 (46.2%)	9 (15.4%) (37.5%)	1 (1.9%) ((4.2%))	3 (5.8%) ((12.5%))	5 (9.6%) ((20.8%))
		Total E4.5 ET	75	13 (17.3%)	4 (5.4%) ((30.8%))	0 (0%) ((0%))	1 (1.3%) ((7.7%))	3 (4%) ((23.1%))
C57BL/6JOlaHsd	KSOM with FGF4	Day3.5 ET	26	11 (42.3%)	10 (38.5%) ((90.9%))	0 (0%) ((0%))	1 (3.8%) ((9.1%))	9 (34.6%) ((81.8%))
		Day4.5 ET	56	3 (5.4%)	3 (5.4%) ((100%)	0 (0%) ((0%))	0 (0%) ((0%))	3 (5.4%) ((100%))
129S2/SvHsd/C57BL/6JOlaHsd		Day3.5 ET	32	11 (34.4%)	10 (31.3%) ((90.9%))	1 (3.1%) ((9.1%))	1 (3.1%) ((9.1%))	8 (25%) ((72.7%))
		Day4.5 ET	30	3 (10%)	3 (10.0%) ((100%))	0 (0%) ((0%))	1 (3.3%) ((33.3%))	2 (6.7%) ((66.7%))
		Total E3.5 ET	58	22 (37.9%)	20 (34.5%) ((90.9%))	1 (1.7%) ((4.6%))	2 (3.5%) ((9.1%))	17 (29.3%) ((77.3%))
		Total E4.5 ET	86	6 (7.0%)	6 (7.0%) ((100%))	0 (0%) ((0%))	1 (1.2%) ((16.7%))	5 (5.8%) ((83.3%))

Efficiency was calculated in relation to the number of injected embryos transferred () and in relation to the number of fetuses retrieved (())

### Pup chimaerism rate improved following exposure to FGF4

**Fig. 4:**
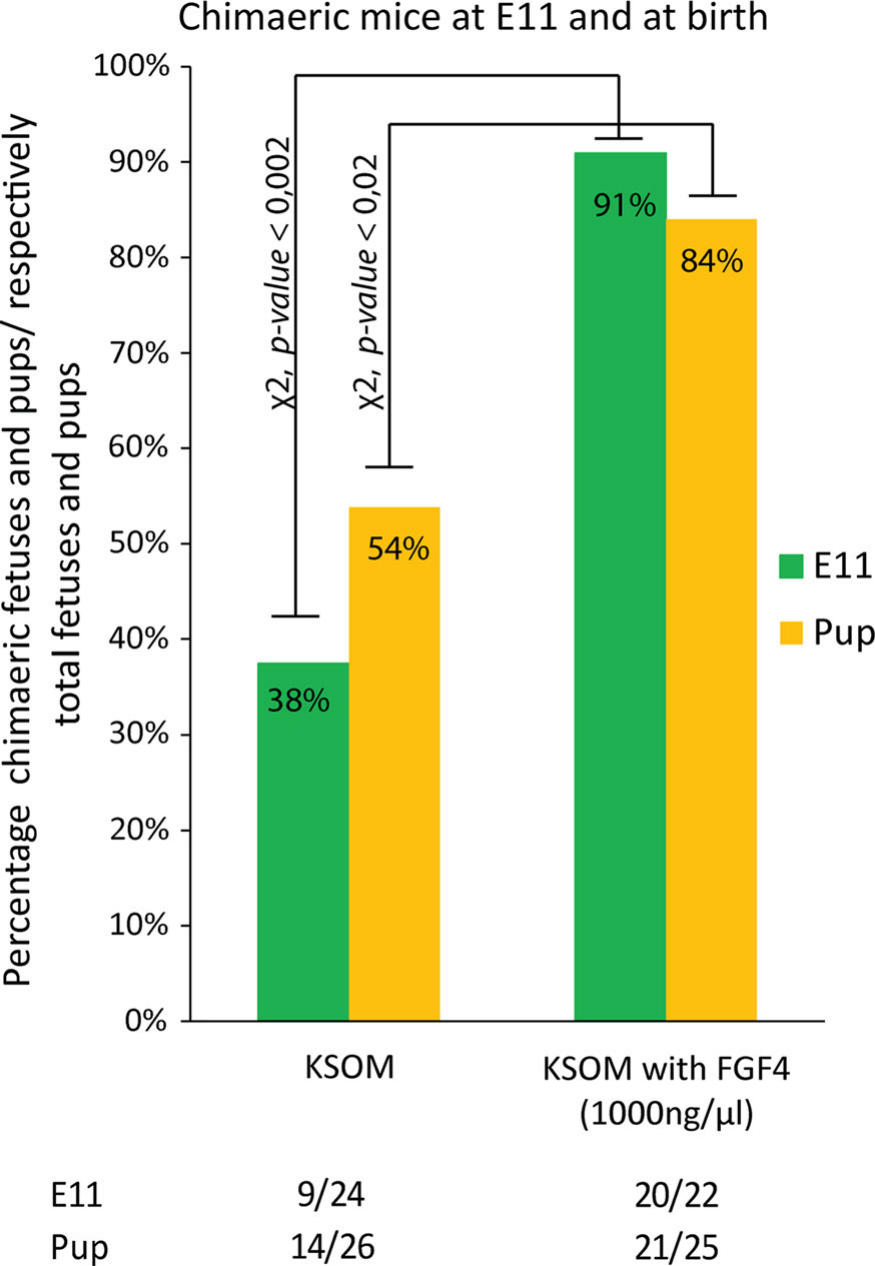
Summary of percentage of chimaeric E11 fetuses and pups. E11 fetuses and pups resulting from blastocysts injected with ES cells (various ES cell lines) respectively compared to the total amount of fetuses and pups. Host embryos were either cultured in KSOM or KSOM supplemented with FGF4 (1000 ng/ml) and heparin (1 µg/ml) prior to and after injection with ES cells on E3.5 and transferred within a few hours (2–8 h) following injection. Significance tested via χ^2^ statistical test

We tested subsequently whether the high proportions of ES cell contribution in E11 fetuses obtained from embryos that were cultured in KSOM supplemented with FGF4 (1000 ng/ml) and heparin (1 µg/ml) also translated in the birth of highly chimaeric offspring. Various hybrid ES cell lines (129S2/SvHsd/C57BL/6JOlaHsd (n = 2), 129S2/SvHsdCastEij and CastEij/C57BL/6JOlaHsd) were injected into embryos that were cultured either in KSOM or KSOM supplemented with FGF4 (1000 ng/ml) and heparin (1 µg/ml). Since previous fetal data had shown that the efficiency per transferred blastocyst is rather low when ET occurs at E4.5, all embryos were retrieved at E2.5 and transferred the same day of the blastocyst injection (E3.5). Significantly more pups, were chimaeric (coat colour) (Table 2) (χ^2^, *p* value <0.02) when transferred blastocysts had been cultured in KSOM supplemented with FGF4 (84%) (1000 ng/ml) and heparin (1 µg/ml) instead of regular KSOM (53.8%). The pups were naturally delivered without c-section and most of them reached adulthood. All the chimaeric mice that were set up for breeding tested positive for germline transmission in the first litter obtained. The percentage of pups relative to the number of transferred blastocysts was lower than reported for the E11 fetuses. Nevertheless, the fetal loss was similar in both tested groups and can therefore not be attributed to FGF4 supplementation to the culture medium. Figure 4 summarizes the percentage of chimaeric fetuses and pups relative to the total amount of fetuses and pups.

**Table 2:** Chimaerisms measured by coat colour

	Treatment		Injected embryos transferred	Total liveborn Pups	Chimaeric liveborn Pups	Chimaeric adult Pups	Germline transmission
129S2/SvHsd/C57BL/6JOlaHsd (n = 2),	KSOM	Day3.5 Transfer	219	22 (10.1%)	11 (5.0%) ((50%))	11 (5.0%) ((50%))	9/9
129S2/SvHsd/CastEij			19	0 (0%)	0 (0%) ((0%))	0 (0%) ((0%))	
CastEij/C57BL/6JOlaHsd			46	4 (8.7%)	3 (6.5%) ((75%))	3 (6.5%) ((75%))	3/3
Total			284	26 (9.2%)	14 (4.9%) ((53.8%))	14 (4.9%) ((53.8%))	12/12
129S2/SvHsd/C57BL/6JOlaHsd (n = 2),	KSOM with FGF4	Day3.5 Transfer	184	17 (9.2%)	13 (7.1%) ((76.0%))	12 (7.1%) ((71.0%))	11/11
129S2/SvHsd/CastEij			18	1 (5.6%)	1 (5.6%) ((100%))	1 (5.6%) ((100%))	ND
CastEij/C57BL/6JOlaHsd			41	7 (17.1%)	7 (17.1%) ((100%))	7 (17.1%) ((100%))	5/5
Total			243	25 (10%)	21 (8.6%) ((84.0%))	20 (8.0%) ((80.0%))	16/16

Efficiency was calculated in relation to the amount of injected embryos transferred () and in relation to the amount of fetuses retrieved (())

**Fig. 5:**
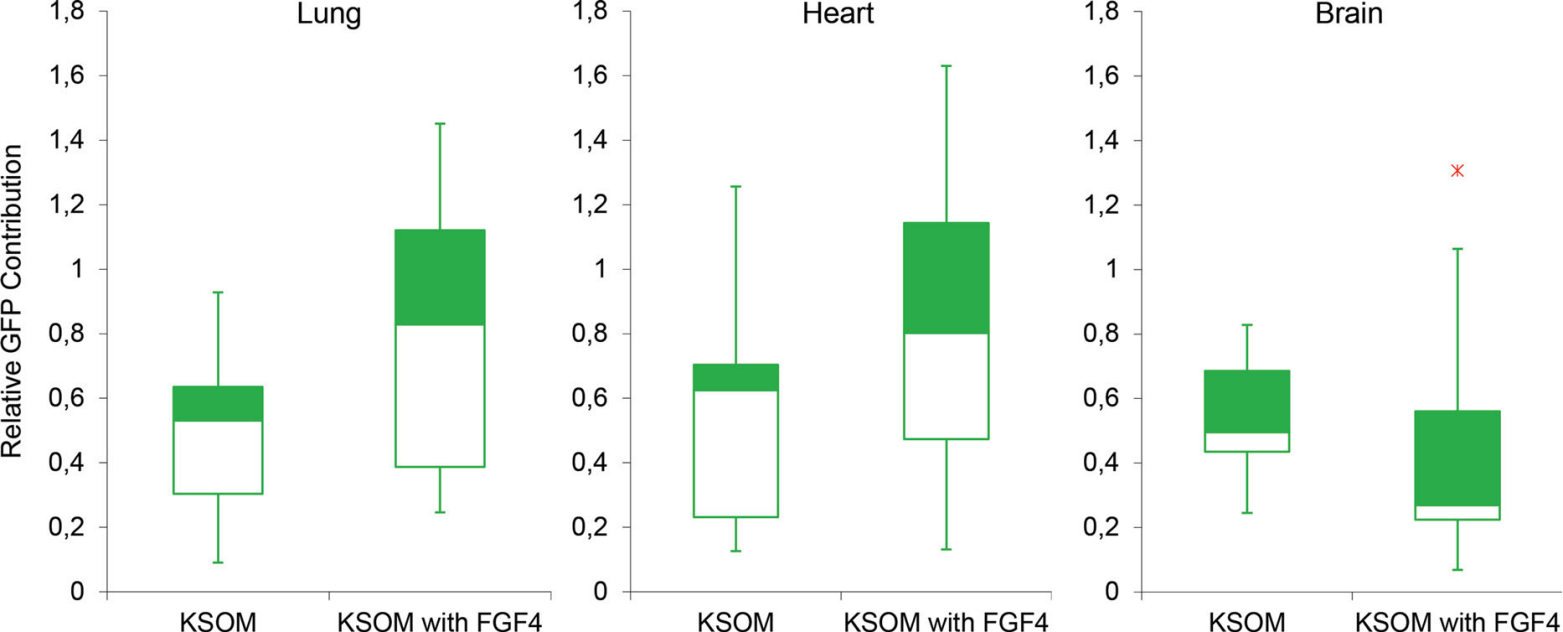
Relative percentage of GFP contribution in tissues. Brain, lung and heart tissues representing ectoderm, endoderm and mesoderm, respectively, in adult chimaeric mice created via ES cell mediated transgenesis either with (n = 15) or without (n = 9) FGF4 and heparin supplementation (x outlier) were assessed for GFP contribution

The contribution of ES cells to offspring (chimaeric as assessed via coat colour contribution) was determined, for those ES cell lines carrying a GFP reporter (129S2/SvHsd/C57BL/6JOlaHsd (n = 1) and CastEij/C57BL/6JOlaHsd), by quantifying GFP contribution in the lungs, heart and brain, As such, 9 chimaeric offspring from the control group were compared to 15 chimaeric offspring from the treated group. The contribution of GFP was estimated by genomic QPCR using primers for GFP and primers for H2A as a reference. For lungs, heart and brain of chimaeric offspring created following the conventional way, the median GFP contribution was 53, 62 and 43%. For offspring derived from blastocysts cultured in KSOM supplemented with FGF4 (1000 ng/ml) and heparin (1 µg/ml) these results amounted to 83, 80 and 23% (Fig. 5). The results show that the rate of chimaerism in lung and heart of adult offspring is higher when host blastocysts were exposed to FGF4 (1000 ng/ml) and heparin (1 µg/ml). Our results obtained with brain, in contrast, show that the high ES cell contribution in lungs and heart cannot be extrapolated to the brain.

## Discussion

The current study provides an alternative method to produce high to near complete chimaeric offspring by directing the fate of the ICM of host embryos towards the primitive endoderm (PE) prior ES cell injection. Two cell populations originate in the ICM, cells that will become the PE or cells that will form the epiblast. According to Yamanaka and colleagues (Yamanaka et al. 2010), the fate of the ICM can be controlled by manipulating the FGF/MAPK/ERK pathway. As such, cells in the ICM of embryos exposed to high doses of FGF4 are biased towards forming the PE, whereas cells in the ICM of embryos exposed to inhibitors of the FGF/MAPK/ERK signaling pathway are biased towards the pluripotent fate. The rate of conversion to primitive endoderm is at its maximum when embryos are exposed to FGF4 levels ranging between 750 and 1000 ng/ml (Yamanaka et al. 2010). In the present study we therefore exposed host embryos for ES cell mediated transgenesis to culture media containing either regular KSOM or KSOM supplemented with FGF4 (1000 ng/ml) and heparin (1 µg/ml) as a cofactor in FGF signaling prior to and after ES cell injection. Since it was shown that the ICM remains responsive to FGF4 or its inhibitors until E4.5 (Yamanaka et al. 2010), we hypothesized that the culture of injected blastocysts into KSOM supplemented with FGF4 (1000 ng/ml) and heparin (1 µg/ml) until the following day might have been beneficial for the rate of chimaerism. Unfortunately, in our hands ET on E4.5 results in a severely reduced viability per transferred embryo. Consequently ETs on E3.5 are preferred. Only embryos that had reformed the blastocoel when replaced in their respective culture medium following injection of ES cells were transferred to uterine horns of pseudopregnant females as we hypothesized that this would favor the rates of chimaerism by reducing the time that the host ICM could revert back to the pluripotent fate.

According to the data from the E11 fetuses, 77% of the fetuses were entirely chimaeric as opposed to 20% in the untreated group. These data are better than the reported numbers of entirely E9 transgenic fetuses conceived following injection of ES cells into pre-compaction preimplantation embryos (Cox et al. 2010). Most chimaeric recovered pups from both experimental conditions displayed a relative high rate of chimaerism as most of them presented a uniform brown colour. The fact that the utilized ES cell lines had a low passage number and were derived and cultured in 2i conditions may provide an explanation for the high contribution efficiency. This high rate also resulted in germline transmission in all chimaeric animals including the animals belonging to the control group. Since the coat colour of the chimaeric mice did not provide discernable differences between both experimental groups, chimaerism was subsequently quantified by establishing the GFP contribution using quantitative RT-PCR. We found that the standard deviation of this last technique was large, and these results should therefore considered as an indicator instead of an exact quantifier. Median values of GFP in lungs and heart contribution approximated 80% when host blastocysts were exposed to FGF4 (1000 ng/ml) and heparin (1 µg/ml) versus 60% in controls. Surprisingly, GFP contribution in brain tissue, which is ectoderm derived, showed the lowest rate of chimaerism with a decreasing trend for animals belonging to the treated group. The latter results are difficult to explain as the coat colour of the mice indicated a high rate of chimaerism and no visual differences in GFP contribution in the head of treated and control E11 fetuses was observed. The reversion of some primitive endoderm biased host ICM cells to the pluripotent fate may explain this finding as these cells are more likely to be found adjacent to the remaining primitive endoderm cells. Indeed, the primitive endoderm forms during further development the visceral endoderm which induces the formation of the neurectoderm from the bordering epiblast cells. Alternatively, the neurectoderm may contain cells with an extra-embryonic origin. ES cell lines carrying specific quantifiable markers should be used in order to quantify the exact rate of chimaerism during subsequent experiments. These experiments would also be helpful to test other ES cell lines in general, since the technique has only been validated for one inbred and four hybrid ES cell lines. In addition, it should be highlighted that the most effective concentration of FGF4 and heparin, needed to bias the host ICM towards PE without compromising viability, will most likely vary depending on the genetic background of the host embryo and should be emprically determined in follow-up experiments.

Chemical induced segregation of the ICM towards PE via FGF4 provides an easy and more feasible alternative to generate mice with high rates of chimaerism in laboratory settings where either tetraploid complementation or ES cell injection into cleavage stage embryos is not effective. Pups can be born via natural delivery and a large percentage of pups are entirely transgenic for tissues from the mesodermal and endodermal lineage. Since the presented technique only relies on biasing cell fate, in contrast to a progressive negative developmental selection of unviable 4 N cells, host embryos in the presented method remain viable. Consequently, whereas no pups will be born via tetraploid complementation if the quality of the utilized ES cells is not good, pups with no or low levels of chimaerism can be born when using the current presented method. Additionally, in contrast to conceptuses created through tetraploid complementation that display high rates of host contamination at implantation (Eakin et al. 2005), the current method provides conceptuses that are merely transgenic from implantation onwards, which makes this technology a very powerful tool to study gene mutations at different stages of embryonic development and in adults.

## References

[CR1] Bradley A, Evans M, Kaufman MH, Robertson E (1984). Formation of germ-line chimaeras from embryo-derived teratocarcinoma cell lines. Nature.

[CR2] Cox BJ, Vollmer M, Tamplin O, Lu M, Biechele S, Gertsenstein M, van Campenhout C, Floss T, Kuhn R, Wurst W (2010). Phenotypic annotation of the mouse X chromosome. Genome Res.

[CR3] Eakin GS, Hadjantonakis AK, Papaioannou VE, Behringer RR (2005). Developmental potential and behavior of tetraploid cells in the mouse embryo. Dev Biol.

[CR4] Eggan K, Akutsu H, Loring J, Jackson-Grusby L, Klemm M, Rideout WM, Yanagimachi R, Jaenisch R (2001). Hybrid vigor, fetal overgrowth, and viability of mice derived by nuclear cloning and tetraploid embryo complementation. Proc Natl Acad Sci USA.

[CR5] Evans MJ, Kaufman MH (1981). Establishment in culture of pluripotential cells from mouse embryos. Nature.

[CR6] Gallagher EJ, Lodge P, Ansell R, McWhir J (2003). Isolation of murine embryonic stem and embryonic germ cells by selective ablation. Transgenic Res.

[CR7] Hu M, Wei H, Zhang J, Bai Y, Gao F, Li L, Zhang S (2013). Efficient production of chimeric mice from embryonic stem cells injected into 4- to 8-cell and blastocyst embryos. J Anim Sci Biotechnol.

[CR8] Kaufman MH, Webb S (1990). Postimplantation development of tetraploid mouse embryos produced by electrofusion. Development.

[CR9] Li XY, Jia Q, Di KQ, Gao SM, Wen XH, Zhou RY, Wei W, Wang LZ (2007). Passage number affects the pluripotency of mouse embryonic stem cells as judged by tetraploid embryo aggregation. Cell Tissue Res.

[CR10] Lu TY, Markert CL (1980). Manufacture of diploid/tetraploid chimeric mice. Proc Natl Acad Sci USA.

[CR11] Martin GR (1981). Isolation of a pluripotent cell line from early mouse embryos cultured in medium conditioned by teratocarcinoma stem cells. Proc Natl Acad Sci USA.

[CR12] Nagy A, Gocza E, Diaz EM, Prideaux VR, Ivanyi E, Markkula M, Rossant J (1990). Embryonic stem cells alone are able to support fetal development in the mouse. Development.

[CR13] Nagy A, Rossant J, Nagy R, Abramow-Newerly W, Roder JC (1993). Derivation of completely cell culture-derived mice from early-passage embryonic stem cells. Proc Natl Acad Sci USA.

[CR14] Okabe M, Ikawa M, Kominami K, Nakanishi T, Nishimune Y (1997). ‘Green mice’ as a source of ubiquitous green cells. FEBS Lett.

[CR15] Poueymirou WT, Auerbach W, Frendewey D, Hickey JF, Escaravage JM, Esau L, Dore AT, Stevens S, Adams NC, Dominguez MG (2007). F0 generation mice fully derived from gene-targeted embryonic stem cells allowing immediate phenotypic analyses. Nat Biotechnol.

[CR16] Shen B, Zhang W, Zhang J, Zhou J, Wang J, Chen L, Wang L, Hodgkins A, Iyer V, Huang X (2014). Efficient genome modification by CRISPR-Cas9 nickase with minimal off-target effects. Nat Methods.

[CR17] Singh P, Schimenti JC, Bolcun-Filas E (2015). A mouse geneticist’s practical guide to CRISPR applications. Genetics.

[CR18] Snow MH (1975). Embryonic development of tetraploid mice during the second half of gestation. J Embryol Exp Morphol.

[CR19] Wang H, Yang H, Shivalila CS, Dawlaty MM, Cheng AW, Zhang F, Jaenisch R (2013). One-step generation of mice carrying mutations in multiple genes by CRISPR/Cas-mediated genome engineering. Cell.

[CR20] Yamanaka Y, Lanner F, Rossant J (2010). FGF signal-dependent segregation of primitive endoderm and epiblast in the mouse blastocyst. Development.

